# Paving the way to a new class of efficient and safe tuberculosis vaccines: The role of c-di-AMP in *Mycobacterium tuberculosis* immunity and virulence

**DOI:** 10.1016/j.omtn.2022.08.034

**Published:** 2022-09-12

**Authors:** Mohamed E. El Zowalaty, Hossam M. Ashour

**Affiliations:** 1 Zoonosis Science Center, Department of Medical Microbiology and Immunology, Uppsala University, Uppsala 75 123, Sweden; 2 Department of Integrative Biology, College of Arts and Sciences, University of South Florida, St. Petersburg, FL, USA

Tuberculosis (TB) remains a challenge and threat to public health despite being a curable and preventable infectious disease. TB is caused by a potentially serious respiratory acid-fast bacillus *Mycobacterium tuberculosis* (Mtb). As one of the top ten causes of death globally, the World Health Organization recently reported that more than 1.5million people died from TB and approximately 10million humans developed TB in 2020.^1^ Until the onset of the coronavirus disease 2019 (COVID-19) pandemic, TB was the leading cause of death from an infectious agent, ranking even before HIV. More dreadfully, it is estimated that approximately 23% of the world’s population (1.7 billion people) have latent TB infections (LTBIs) and that 10% of people with LTBIs will eventually develop TB disease.^1^

Among the current challenges of TB treatment and prevention are the limited number of antimycobacterial agents available for clinical use and the emergence of spreading, multiple, and extremely drug-resistant Mtb. In addition, the only approved TB vaccine is the current vaccine Bacille Calmette-Guérin (BCG), which provides partial and variable protection. Currently, there are at least 16 candidate vaccines in various stages of clinical development in the global TB vaccine pipeline, as was reviewed elsewhere.^2^^,^^3^

TB prevention measures have relied on the use of BCG for more than 100 years since its first use in 1921. Compounding the urgency of the global TB epidemic and looming pandemic fears, there is an urgent need for novel and highly efficient TB vaccines for different age groups including adolescents and adults to help prevent transmission and eventually end TB.

The detection of the bacterial second messenger, cyclic-di-adenosine monophosphate (c-di-AMP) by the host cytoplasmic surveillance pathway (CSP) is known to elicit type I interferon (IFN) responses and autophagy, innate immune responses that are crucial to antimicrobial defense, and modulation of this small molecule may lead to the development of novel immunotherapeutic-based strategies against TB.^4^

The role of bacterial c-di-AMP in the virulence or induction of innate immune responses against Mtb was recently investigated;^4^^,^^5^ however, the role of mycobacterial c-di-AMP in the efficacy and level of attenuation of TB vaccine candidates has been poorly understood and is still unclear.

In this issue of *Molecular Therapy – Nucleic Acids*, the study by Perez et al.^6^ contributes to our understanding of how the Mtb PhoPR virulence system negatively regulates the synthesis of c-di-AMP. Double deletions of phoP and fadD26 in the live-attenuated TB vaccine candidate Mtb vaccine (MTBVAC) produced a several-fold increase in c-di-AMP levels relative to wild-type Mtb or the currently used vaccine BCG.

In addition to a more efficacious vaccine response, Perez et al. also found that c-di-AMP contributed to a safer profile of live TB vaccine in an immunodeficient mouse model.^6^ They demonstrated that the overproduction of c-di-AMP due to inactivation of cnpB resulted in lower protection of MTBVAC, while the absence of c-di-AMP in the MTBVAC disA derivative maintained the protective efficacy of this vaccine in mice.

Upon infection of human macrophages, the MTBVAC derivatives generated significantly higher levels of the proinflammatory cytokine interleukin-1β (IL-1β) responses compared with BCG when tested *in vitro*. However, the IFNβ responses, which include anti-inflammatory effects, were muted.

It would be crucial to measure IL-10 and IL-4 responses to assess the involvement of the Th2 response and the impact on immunoregulation. It would be timely to finally address the understudied and underappreciated roles of B cell antigen presentation and B cell-mediated antibody production in the context of vaccination against TB and in the context of protection against TB infection. Moreover, it would be prudent to measure and understand how antibody levels correlate with the administration of the candidate vaccine and if a correlation exists between a robust T cell response to vaccination and protective and neutralizing antibody response.

One consideration with the candidate vaccine is the potential maintenance of a pro-inflammatory environment. This can lead to chronic inflammation and possibly cytokine release syndrome (CRS) or CRS-like responses, which have been previously observed with chimeric antigen receptor (CAR) T cell therapy and with mRNA vaccines. A proper balance between pro- and anti-inflammation needs to be maintained so that inflammation does not lead to dire health consequences.

Since autoantibodies have already been observed in the blood of patients with mycobacterial infections, it would be important to ensure that any candidate *Mtb* vaccine does not stimulate further autoimmunity in patients. Other considerations include the choice of a proper route of administration that maximizes the protective immune responses to the candidate vaccine. Thus, a careful choice between oral, intradermal, intravenous, intranasal, aerosol, and pulmonary routes needs to be investigated.

The ability of a small second messenger molecule to control the consequences of intracellular infection strongly suggests new approaches to develop new therapeutics and prevent TB and related challenging and difficult-to-control infections. The modulation of c-di-AMP may have the potential to pave the way toward the development of licensed, safer, and more efficient TB vaccines for clinical application, which will help prevent TB and end the global TB epidemic.
